# Extensive pyomyositis of vastus muscles

**DOI:** 10.11604/pamj.2017.28.30.13544

**Published:** 2017-09-14

**Authors:** Chong Yau Ong, Jin Lee Lim, Lourdes Ducusin Galang

**Affiliations:** 1 Chong Yau Ong, Department of Family Medicine and General Medicine, Sengkang General Hospital, 378 Alexandra Road, 159964 Singapore; 2 Department of Internal Medicine, Hospital Sultanah Aminah, Malaysia; 3 Department of Internal Medicine, Singapore General, Hospital Sultanah Aminah, Singapore

**Keywords:** Pyomyositis, thigh, abscess, staphylococcus aureus

## Abstract

We report a case study on a patient who presented with low back and thigh pain of one month duration. He was eventually diagnosed with left thigh pyomyositis. Tissue from thigh grew Staphylococcus aureus. With commencement of antibiotics and surgical drainage, patient made recovery despite prolonged hospital stay. The underlying mechanism of the extensive abscess accompanied by lack of systemic symptoms; is related to relative immunocompromised state of having underlying diabetes mellitus.

## Introduction

Pyomyositis is an acute bacterial infection occurring in the skeletal muscle with localized abscess formation [1]. Skeletal muscles are generally resistant to infection; however infection can take place in the presence of predisposing factors. Pyomyositis is comparatively rare in the temperate climates compared to tropical although there has been more reports from the temperate climates over the past decades. Increased in reports from temperate regions could be due to combination factors of increased awareness, improvement in diagnosis and increased in the number of immune-compromising conditions. Pyomyosistis is most commonly caused by Staphylococcus aureus (90%) followed by Group A Streptococci (1-5%) [2]. Other rarer organisms implicating pyomyositis are Streptococcus (group B, C, G), pneumococcus, Neisseria spp, Haemophilus spp, Aeromonas spp, Klebsiella spp, Yersenia spp, Pseudomonas spp and Escherichia spp [3, 4]. Most commonly affected muscles are the lower extremities (quadriceps, gluteal and calves) although psoas muscles, iliacs, pectoralis major, stenocleidomastoid and upper extremities muscles involvement have been reported [1, 5, 6]. We present a case of extensive Staphylococcal pyomyositis with relatively non-specific presentation thus causing diagnostic challenge, in which could have been clinched earlier. In view of its relative rarity and ambiguous presentation, physicians should have heightened awareness of this disease, especially in the context of immunocompromised patients.

## Patient and observation

A 62-year old Chinese man presented to the emergency department with complaints of low back pain and left thigh pain for one month. He has a background of hypertension and gout, on no medications. There was no trauma or injury to the back. He was able to walk. There was no fever. Initial examination on first presentation revealed normal examination of the back and left thigh (no swelling or erythema or tenderness noted. Lumbar X-ray imaging showed spondylotic changes of L4-5; femur X-Ray imaging showed no fracture and vascular calcifications. Full blood counts (FBC) showed a white blood cell count of 11.9 (X10(9)/L) with 70.4% neutrophils. Creatine kinase was normal at 48 U/L and renal panel was unremarkable except for random glucose was 11.7 mmol/L. He was discharged with topical and oral analgesics. He was reviewed again at the orthopedic clinic nine days later and was given other oral analgesics in because of his persistent left thigh pain; which was documented to be tender but no other changes were noted. Three weeks later patient attended physiotherapy session. The left thigh was felt to be warm and with mild tenderness. Thigh circumferences were 35cm on the right, 40cm on the left (taken just above patella), right 35cm and left 41cm (taken 5cm above patella). Calves were supple with bilateral pedal edema. Gait training using broad base quad stick was prescribed and cryotherapy to warm area was advised; with a memo to doctor to reevaluate the non-resolving pain and with left thigh swelling. Patient however did not seek the doctor and returned two weeks later to the scheduled physiotherapy session. His left thigh was still 40cm in circumference (5cm above patella) and warm. He was referred to the accident and emergency department.

**Figure 1:**
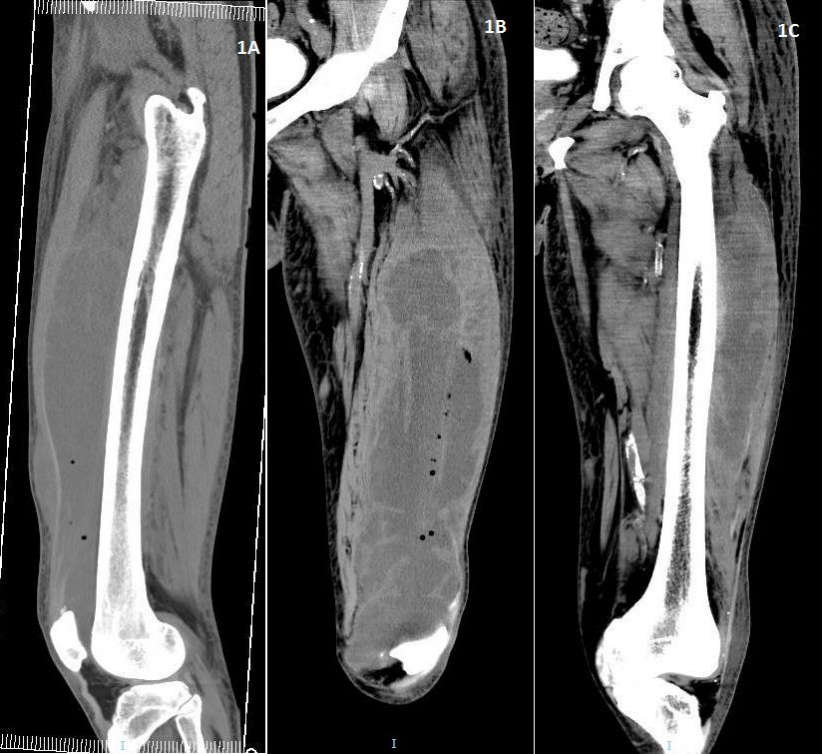
(A) AP medial view; (B) posterior view; (C) (posterior view) Longitudinal sections shows right thigh enlargement with fluid collection and scattered air pockets within vastus lateralis. There was extensive involvement from the lesser trochanter down to the left knee joint level

**Figure 2:**
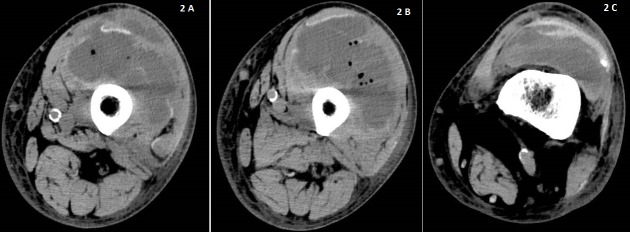
(A, B, C) cross sectional images of the thigh from superior to inferior (femoral condyles seen). Fluid collection and air bubbles are again seen in the anterior compartment

In the accident and emergency, the initial impression was deep vein thrombosis to rule out pulmonary embolism as our patient revealed a travel history to China and back to Singapore (four hours flight) six weeks ago. There was no trauma or injury. There was no shortness of breath and chest pain. There was no fever. He was afebrile (T 36.8^o^C) but was tachycardic with heart rate of 105 beats per minute. Blood pressure was 161/71 mmHg and respiratory rate of 20 breaths per minute with oxygen saturations of 95% under room air. Examinations revealed decreased air entry on the left middle and lower zones of the lungs. Distal pulses were well felt. D-dimer quantitation was elevated at 4.86 (< 0.05mg/L FEU). White blood cells were elevated at 26.04X10^9^mg/L). Patient was admitted the same day in the evening for further work up of the above. CRP was 221 MG/L and procalcitonin level was 0.87 UG/L (< 0.5 UG/L). Blood cultures was taken and he was started on empirical intravenous cefazolin 2g every eight hourly. Chest X-Ray (AP film) revealed minimal air space shadowing in the lung bases. A repeated morning blood showed down trending of white blood cells at 21.23; however procalcitonin remained elevated at 1.2 UG/L. Urinalysis showed pyuria. Clinically he was less tachycardic with heart rate of 94-98 bpm; maintaining good saturations on room air with normal blood pressure and was afebrile. Antibiotic was switched to intravenous ceftriaxone 2g daily and metronidazole 500mg every eight hourly for sepsis likely urinary or intraabdominal source. CT chest, abdomen and pelvis revealed bilateral pleural effusions with patchy consolidations of upper lobes and no intraabdominal collection. The left common iliac lymph nodes were found to be enlarged. The partially imaged superior section of thigh showed mildly hyperdense lesion in the anterior segment of left thigh. Possibilities include a soft tissue lesion or haematoma. Ultrasound Doppler of the left lower limb showed no evidence of deep vein thrombosis. Edema was noted in the subcutaneous tissue of the left thigh. CT scan of the left thigh subsequently showed large left vastus muscles intra-muscular fluid collection with air bubbles (Figure 1, Figure 2).

Patient underwent incision and drainage of the abscess. 800ml of blood-stained pus intramuscularly in the left thigh contained in a sac involving vastus lateralis and vastus intermedius was drained. No dishwater fluid along fascia. Muscles were friable but contracts to diathermy. Post operatively our patient had hypovolemic shock from intraoperative blood loss. This was responsive to fluid resuscitation and blood transfusions. Three sets of initial blood cultures (aerobic and anaerobic) taken since admissions were negative. Urine culture on admission was negative. Fluid culture grew Staphylococcus aureus, sensitive to penicillin, ampicillin, cloxacillin, cefazolin, cotrimoxazole, clindamycin and erythromycin. Two days later, patient underwent left thigh wound debridement. The wound bed was clean and there was no pus. Superficial muscles were viable. Seven days after the initial debridement, another wound debridement was done with partial wound closure. Following this, a secondary closure of wound was done two days later. All three sets of tissue cultures grew methicillin sensitive Staphylococcus aureus (MSSA). Our patient was transferred to a community hospital for rehabilitation and for continuation of IV Cefazolin after eighteen days of stay in general hospital (as he developed congestive cardiac failure). He turned up for subsequent follow ups. He was seen at orthopedic clinic one month post operation. He was ambulating and wounds healed. He was given open date follow up. He has attended physiotherapy sessions and improvement noted as he was able to be weaned off broad based quad stick and back to independent ambulation without aid.

## Discussion

Our patient had a less typical presentation of pyomyositis without pyrexia. The insidious onset of symptoms with only pain without fever rendered the clinical diagnosis challenging and easily missed out [2]; which happened in the first two consults. Physicians need to note that any infection, including bacteremia can present in the absence of fever. One study reported temperature of > 38 degree Celsius in only 59% of the patients [4]. Diagnosis is often delayed by two to three weeks. Although he has no erythema (rubor) on the thigh area, he presented with all other three cardinal signs of inflammation-pain (dolor), swelling (tumor) and heat (calor). There is no fluctuance as the abscesses are deep seated in the muscles unlike superficial skin infections. Presence of the above should trigger suspicion for muscle infections in the setting of immunocompromised host. Apart from the atypical presentation, the delayed arrival to the diagnosis is also attributed to the consideration of other primary diagnoses; which by far are more common [7]. These conditions include muscle strain, contusion, cellulitis, hematoma, deep vein thrombosis, septic arthritis and soft tissue sarcoma. Our patient has a diabetes mellitus which was newly diagnosed when he presented with pyomyositis. It is not uncommon for diabetics to be first presented with acute infections such as urinary tract infection and superficial skin infections such as cellulitis and carbuncles. Impaired tissue perfusion and impaired chemotaxis, phagocytosis and bactericidal activity of neutrophils are some of the known mechanism of defective immune defense among diabetics. Other predisposing factors for pyomyositis is immune-related conditions such as use of corticosteroids, human immunodeficiency syndrome, malignancies and intravenous drug abusers [2, 8–10]. There are three stages of evolution of pyomyositis: the invasive stage, purulent stage and late stage. The invasive stage is typically sub-acute (occurring over one to three weeks) whereby seeding of the bacteria in the muscle takes place. It is clinically insidious with local swelling, mild pain and variable fever. In the purulent stage (usually 10 to 21 days), physical signs are more apparent and this is the stage where diagnosis are usually made. In the late stage, complications such as rhabdomyolysis, septic shock and other manifestations of disseminated infection occur. Pyomyositis at this stage can be life threatening.

Laboratory findings are not specific for pyomyositis. Leukocytosis and an increase in the erythrocyte sedimentation rate (ESR) are often observed. However this may not be present in patients with neutropenia or immunodeficiency state. Interestingly, creatine phosphokinase and aldolase levels is found to be normal in pyomyositis [1, 11]. Blood cultures are positive in only 5% to one-third of patients while positive culture of aspirated pus has a high yield of 70% to 85% [1, 3, 12]. Both computed imaging (CT) and magnetic resonance imaging (MRI) are choice radiological studies in establishing diagnosis [13]. CT of abdomen is able to look for abscess and collections in the abdomen as intra-abdominal sepsis can lead to secondary thigh abscesses [14]. Although the presence of air bubbles in the tissues were unusual in staphylococcal infection; there has been report of Staphylococcus abscess with gas bubbles [15]. There was no prior aspiration and only Staphylococcus was cultured from the tissue specimens. MRI if available, is the most precise technique to use in determining the exact location and the extent of the disease [16]. Hyper intense signal can be detected in T2-weighted images in early stages, while abscess with rim enhancement (post contrast) is seen in late stage of the disease. Ultrasonography as diagnostic modality is increasingly helpful in settings of emergency units and restricted resources whereby CT and MRI are not readily available. Successful management of abscess includes early recognition of focus of infection, timely surgical debridement or drainage, resuscitation if required and appropriate antibiotic therapy [17]. Draining thigh abscess from thigh incision allows direct approach and allows assessment of viability of muscle and fascia of the thigh, as well as the need for further debridement [18]. Miller et al in their observational study had found that lack of incision and drainage was associated with nonresponse post discharge, although there was no significant in response rate observed in between MRSA and MSSA groups [7, 19]. In countries with risk and emergence of community acquired methicillin-resistance Staphylococcus aureus, vancomycin is recommended as the first choice of antibiotic therapy [8, 11]. The duration of antibiotic therapy depends on clinical improvement. Primary pyomyositis without bone or joint involvement can be treated with total duration of three weeks (initially with intravenous and then switching to oral agent after clinical improvement) [1, 8].

## Conclusion

Pyomyositis can be insidious in onset and easily missed out by unwary physicians. Heightened awareness of this condition is important so that early intervention can be instituted.

## Competing interests

The authors declare no competing interests.
